# IgG4-related membranous glomerulonephritis and generalized lymphadenopathy without pancreatitis: a case report

**DOI:** 10.1186/s12882-017-0561-2

**Published:** 2017-04-26

**Authors:** Justine Huart, Stéphanie Grosch, Christophe Bovy, Michel Moutschen, Jean-Marie Krzesinski

**Affiliations:** 1 Division of Nephrology, University of Liège Hospital (ULg CHU), Liège, Belgium; 2 Division of Infectious diseases and General Internal Medecine, University of Liège Hospital (ULg CHU), Liège, Belgium

**Keywords:** IgG4-related disease, IgG4-related kidney disease, Lymphadenopathy, Membranous glomerulonephritis, Nephrotic syndrome

## Abstract

**Background:**

IgG4-related disease is a recently described pathologic entity. This is the case of a patient with nephrotic syndrome and lymphadenopathy due to IgG4-related disease. Such a kidney involvement is quite peculiar and has only been described a few times recently. Renal biopsy showed a glomerular involvement with membranous glomerulonephritis in association with a tubulo-interstitial nephropathy. Moreover, the patient was not suffering from pancreatitis.

**Case presentation:**

The patient is a middle-aged man of Moroccan origin. He has developed recurrent episodes of diffuse lymphadenopathies, renal failure and nephrotic syndrome. Renal biopsies showed membranous glomerulonephritis.

**Discussion and conclusion:**

The diagnostic approach of this atypical presentation is discussed in this case report as well as diagnostic criteria, therapeutic strategies, biomarkers and pathophysiology of IgG4-related disease. IgG4-related membranous glomerulonephritis is a well-established cause of membranous glomerulonephritis. It must be sought after in every patient with a previous diagnosis of IgG4-related disease and in every patient with this histological finding on renal biopsy. Corticoids are still the first-line treatment of IgG4-related disease. New therapeutic strategies are needed to avoid glucocorticoids long term side-effects. Interestingly, the patient was prescribed cyclophosphamide in addition to glucocorticoids for an immune thrombocytopenia. This treatment had a very good impact on his IgG4-related disease.

## Background

IgG4-related disease (IgG4-RD) was first described 50 years ago as a pancreatitis with hypergammaglobulinemia and called autoimmune pancreatitis (AIP). The immunoglobulins involved in this AIP were thereafter characterized as belonging to the IgG4 subclass. Multisystemic involvement of the disease was noticed only in 2003 and the name IgG4-RD finally chosen to designate this pathology. All denominations previously used such as “IgG4 syndrome” or “IgG4-related sclerosing disease were subsequently abandoned. Moreover, new denominations were established for each organ involved i.e. “IgG4-related kidney disease” (IgG4-RKD) for kidney involvement. Therefore, several diseases named before the knowledge of their belonging to the IgG4-RD entity had to be renamed. For example, “IgG4-related pancreatitis” is now the correct name for AIP, as is “IgG4-related dacryoadenitis and sialadenitis” the correct name for Mikulicz syndrome [1, 2].

IgG4-RD is more common in middle-aged men and can involve every organ. Tumefactive lesions are characteristic of the disease with pseudotumoral swelling often being its first clinical manifestation. Organ dysfunction, imaging or biopsic findings can also reveal IgG4-RD. The histological pattern of lesions is fibro-inflammatory with a lymphoplasmocytic infiltrate enriched with IgG4+ plasma cells and storiform pattern of fibrosis. Obliterative phlebitis and eosinophilic infiltration are also commonly observed. The degree of fibrosis depends on the tissue involved and increases with time. Marked fibrosis is often described in IgG4-related retroperitoneal fibrosis (previously called Ormond’s disease) and in IgG4-related thyroid disease (previously Riedel’s thyroiditis). Pancreatitis is the most common manifestation of IgG4-RD and is often associated with lymphadenopathy, salivary glands and kidney involvement. IgG4-RD without pancreatitis is much less common [1, 3].

## Case presentation

### Patient’s clinical history, explorations and treatment (Table 1)

**Table 1:** Main laboratory work-up, imaging and histology studies from 2004 to 2012

Year	Investigations	Results
2004	*Laboratory Work-up*	
	Blood tests	Serum creatinine: 3.51 mg/dL, eGFR: 24 ml/min/1.73 m^2^, low serum C3 and C4 levels, hypergammaglobulinemia (4590 mg/dL), hypoalbuminemia, negative ANA.
	Urine tests	Proteinuria (13800 mg/l).
	*Imaging studies*	
	Kidney ultrasound	Diffuse kidney enlargement.
	Abdominal MRI	Infracentimetric lombo-aortic lymphadenopathies and kidney enlargement.
	PET/CT	Sub and super diaphragmatic hypermetabolic lymphadenopathies compatible with lymphoproliferative disorder associated with heterogeneous fixation in kidneys.
	Whole skeleton X-Ray	No lesion.
	*Histology studies*	
	Bone marrow biopsy	Suspicion of myelodysplastic syndrome.
	Lymph node biopsy	Reactive lymphoid hyperplasia.
	Renal biopsy	MGN with IgG and C3d deposits associated with severe TIN and lymphoplasmocytic infiltrate.
2005	*Histology studies*	
	Skin biopsy	IgG and C3d deposits.
2008	*Laboratory Work-up*	
	Blood tests	Serum creatinine: 1.55 mg/dL, eGFR: 51 ml/min/1.73 m^2^, low serum C3 and C4 levels, hypergammaglobulinemia (3460 mg/dl), ANA (1/80) characterized as anti-dsDNA, thrombocytopenia.
	Urine tests	Proteinuria (6081 g/l).
	*Imaging studies*	
	Abdominal CT	Iliac and aortic supra-centimetric lymphadenopathies.
	PET/CT	Sub and super diaphragmatic hypermetabolic lymphadenopathies in progression compared to 2004.
	Whole skeleton X-Ray	No lesion.
	*Histology studies*	
	Bone marrow biopsy	Reactive lymphoid hyperplasia of bone marrow.
	Lymph node biopsy	Reactive lymphoid hyperplasia.
	Renal biopsy	MGN with IgG and C3d deposits associated with chronic interstitial nephropathy (mononuclear infiltrate) without clear sign of tubular involvement.
2012	*Laboratory Work-up*	
	Blood tests	Serum creatinine: 1.6 mg/dL, eGFR: 48 ml/min/1.73 m^2^, low serum C3 and C4 levels, hypergammaglobulinemia (4250 mg/dL) with oligoclonal banding, ANA (1/640), Anti-dsDNA negative, IgG4 (2790 mg/dL), negative PLA2R antibodies test.
	Urine tests	Proteinuria (444 mg/l).
	*Imaging studies*	
	Thoraco-abdominal CT	Sub and super diaphragmatic supracentimetric lymphadenopathies.
	PET/CT	Sub and super diaphragmatic hypermetabolic lymphadenopathies in progression compared to 2008.
	*Histology studies*	
	Lymph node biopsy	IgG4-related lymphadenopathy (IgG4+/IgG+ cells > 40% and >10 IgG4+ plasma cells per HPF).

Legend: *ANA* anti-nuclear antibodies, *CT* computed tomography, *HPF* high powered field, *MGN* membranous glomerulonephritis, *MRI* magnetic resonance imaging, *PET/CT* positron-emission tomography/computed tomography, *PLA2R* anti-phospholipase A2 receptor, *TIN* tubulo-interstitial nephropathy

**Fig. 1:**
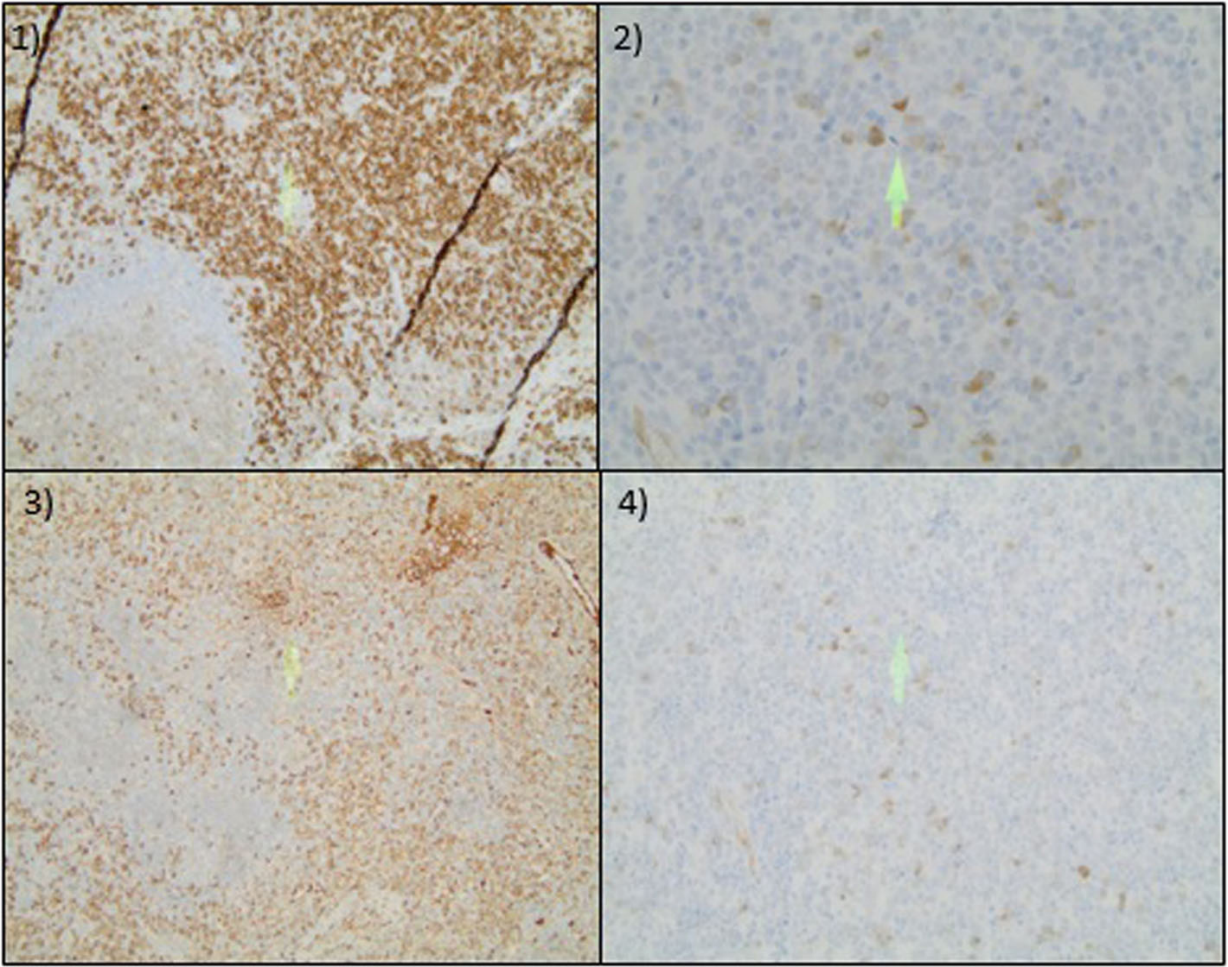
Lymph node biopsy: histopathological diagnostic criteria for IgG4-RD. 1) Highlighting of plasma cells with CD38 × 20: *brown* cells are positive for CD38 which indicates most cells in the lymph node are plasma cells. Eosinophilic infiltration is also present. 2) Highlighting of IgG4+ plasma cells × 40. *Brown* cells are IgG4+ plasma cells. There are more than 10 IgG4+ plasma cells on the picture that represents a high powered field. 3) Highlighting of IgG+ plasma cells × 20. 4) Highlighting of IgG4+ plasma cells × 20. More than 40% of IgG+ plasma cells are estimated to be IgG4+

A 40-year old male patient of Moroccan origin was admitted in 2012 for diffuse lymphadenopathy confirmed with ultrasound. Blood tests revealed renal insufficiency (serum creatinine: 1.6 mg/dL, eGFR: 48 ml/min/1.73 m^2^), low serum C3 and C4 levels and hypergammaglobulinemia (4250 mg/dL with oligoclonal banding). Anti-nuclear antibodies (ANA) were positive at 1/640. Anti-dsDNA were negative. Urine tests showed proteinuria (444 mg/L). A thoraco-abdominal computed tomography and positron-emission tomography/computed tomography (PET/CT) were performed. The images were compatible with a lymphoproliferative disorder. Heterogeneous uptake was also noticed in kidneys. Lymph node biopsy was performed and ruled out a hematologic malignancy. Immunohistochemistry showed more than 10 IgG4+ plasma cells per high powered field (HPF) of biopsy sample with more than 40% of IgG+ plasma cells being IgG4+ (Fig. 1). Serum IgG4 level was then measured at 2790 mg/dL (*N* < 201 mg/dL).

**Fig. 2:**
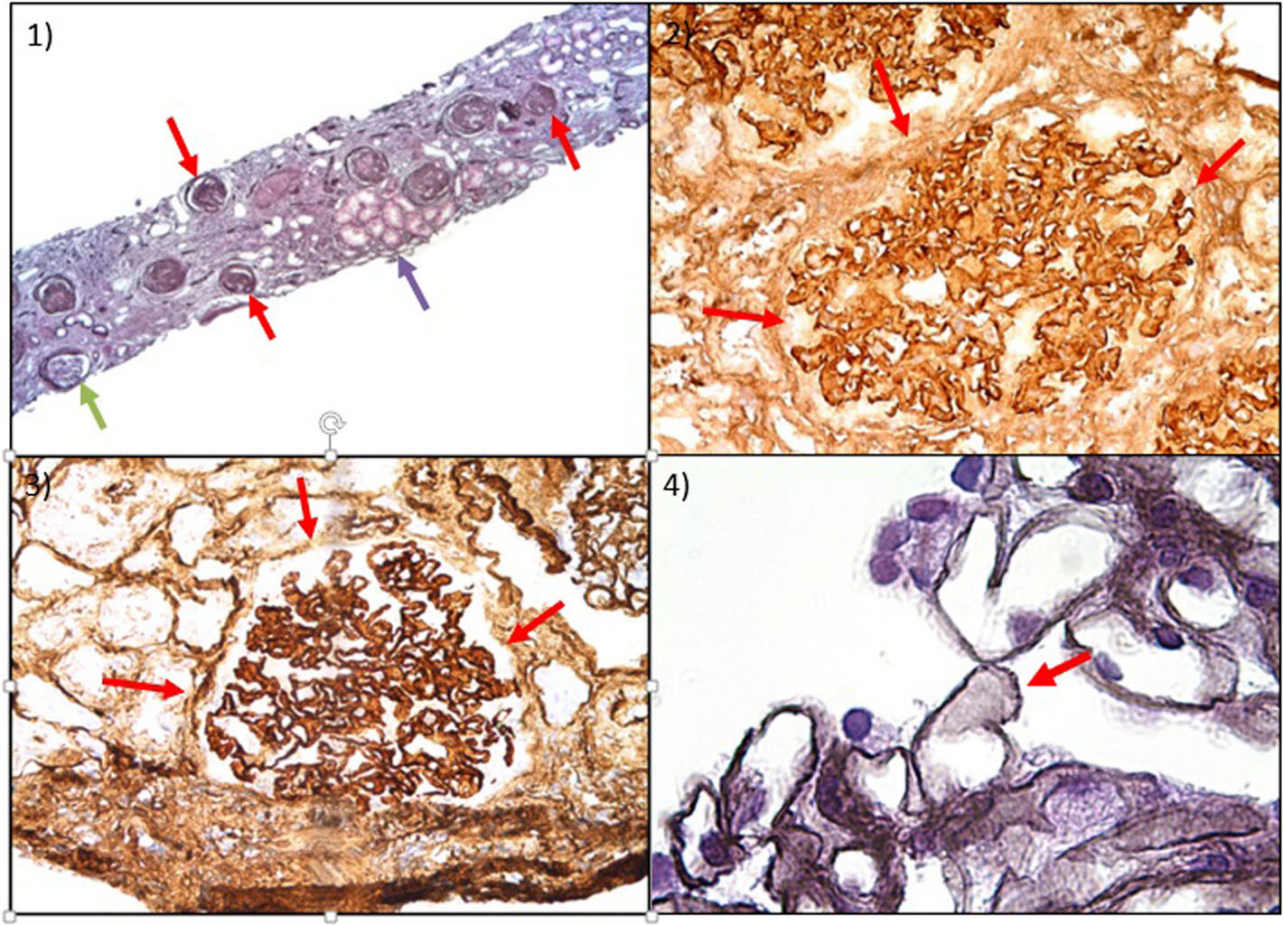
Renal biopsy: membranous glomerulonephritis and highlighting of IgG and C3d immune complexes’ deposits. 1) Hematoxylin Eosin × 4: *red* arrows show abnormal glomeruli. *Green* arrow shows normal glomerulus (nevertheless capsular fibrosis is observed). *Purple* arrow shows normal tubuli. 2) Highlighting of IgG’s deposits × 20: *red* arrows surround pathological glomerulus. IgG’s deposits in this glomerulus show more contrast than surrounding tissues. 3) Highlighting of C3d immune complexes’ deposits × 20: *red* arrows point to pathological glomerulus which shows more contrast than surrounding tissues because of C3d immune complexes’ deposits. 4) Immersion M + × 100: *red* arrow shows membranous glomerulonephritis’s typical thickening of glomerular basement membrane. This thickening is due to reaction against IgG and C3d’s deposits

This episode was not the first in patient’s medical history. In 2004, the patient had been admitted for lower-limb edemas and diffuse lymphadenopathy. Renal insufficiency (serum creatinine: 3.51 mg/dL, eGFR: 24 ml/min/1.73 m^2^), low serum C3 and C4 levels and hypergammaglobulinemia (4590 mg/dL) had already been found. Urine tests showed high proteinuria (13800 mg/l) with hypoalbuminemia characteristic of a nephrotic syndrome. Renal ultrasound showed diffuse kidney enlargement. PET/CT showed the same results as the one performed in 2012. A lymphoproliferative disorder was excluded by whole skeleton X-Ray, bone marrow and lymph node biopsy. A renal biopsy was then performed and showed membranous glomerulonephritis (MGN) with IgG and C3d deposits (Fig. 2). Severe tubulo-interstitial nephropathy (TIN) with lymphoplasmocytic infiltrate (CD20+, CD3+, CD5+) was associated. Secondary causes of MGN were rejected by appropriate tests. ANA were negative at the time. The proposed diagnosis was a dysimmune state of unknown origin with secondary MGN and reactive lymphadenopathy. Treatment with glucocorticoids (GC) was started with an excellent clinical and biological response.

In 2005, a cutaneous rash appeared on the left arm and was biopsied, showing IgG and C3d deposits. GC were stopped in 2006 given the recovery of renal function. A few months later, the patient started to complain of pain, redness and swelling in small joints of the feet and hands.

In 2008, the first relapse occurred with the same clinical picture that in 2004 (nephrotic syndrome and lymphadenopathy). Full assessment was repeated yielding similar results except for renal biopsy: MGN with IgG and C3d deposits was found again but chronic interstitial nephropathy replaced the severe TIN. Indeed, a mononuclear infiltrate was present in the interstitium without clear sign of tubular involvement. A severe thrombocytopenia (platelets: 8.10^3^/mm^3^) developed a few weeks later with anti-dsDNA found on blood sample (1/80). Thrombocytopenia was refractory to perfusion of immunoglobulins, GC and cyclophosphamide (CYP). Plasmapheresis and splenectomy were then performed. CYP was next continued for 6 cycles (500 mg/cycle) with good efficacy. Relapsing thrombocytopenia was successfully treated by additional regiments of immunoglobulins and GC. The last cycle of CYP was completed at the end of 2008. Thereafter, the patient experienced a long period without any problem until the next hospitalization in 2012.

**Fig. 3:**
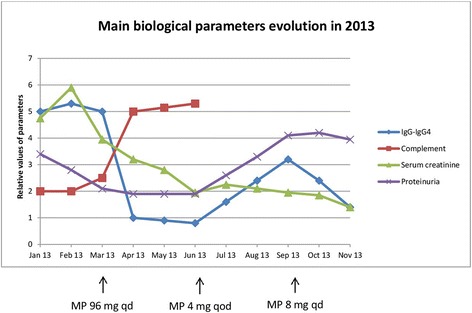
Graphical representation of main biological parameters evolution in 2013. At the beginning of the year, patient experienced a new relapse of his IgG4-RD with impairment in renal function (increase in serum creatinine) and an increase in proteinuria. A lowering of serum complement level is noticed as well as increased IgG and IgG4 levels at that time. Induction GC therapy started in march and one notices from that moment on a diminution of IgG-IgG4 level as well as an increase of serum complement level while renal function improved (normalization of serum creatinine). Around July, one sees a new increase in IgG and IgG4 levels which is consistent with dose reduction in GC therapy. New impairment in renal function was avoided this time by increasing daily dose of GC. MP = methylprednisolone; qd = daily; qod = every other day

In 2012, a renal biopsy was not performed again despite a new relapse of nephrotic syndrome; indeed, the 2008 biopsy had led to a macroscopic urinary hemorrhage which had required embolization. MGN was assessed again and anti-phospholipase A2 receptor (PLA2R) antibodies were negative. Diagnosis of IgG4-RD was finally made on the basis of the lymph node biopsy, with IgG4-related generalized lymphadenopathy and IgG4-related MGN (associated with IgG4-related TIN in 2004). The patient was given GC again at high dose (methylprednisolone 96 mg daily) for 1 month followed by a progressive decrease of the dose. Rituximab use was discussed in 2013, due to a new relapse in spite of maintenance low dose GC therapy and development of side effects; unfortunately, the patient failed to obtain the reimbursement and the medication could not be given in this context. For now, the patient is well stabilized with low dose GC. Rituximab in compassionate use could be reconsidered in case of a new relapse. Figure 3 shows the evolution of various biological markers and serum creatinine during 2013.

## Discussion

### Discussion about patient’s diagnosis

This atypical presentation of a rare pathology (i.e. the patient never experienced any episode of pancreatitis) made difficult the diagnosis of IgG4-RD. Moreover, IgG4-RD was not well known at the time of the first symptoms in 2004 and PLA2R antibodies were not available in daily practice at that time.

First of all, hematologic malignancies had to be excluded in the presence of lymphadenopathy and biological abnormalities. Moreover, a higher risk of developing lymphoma or neoplasia is highly suspected in IgG4-RD patients [4]. Other systemic diseases such as Sjögren’s syndrome, sarcoidosis and especially systemic lupus erythematous (SLE) were also part of the differential diagnoses. A diagnosis of SLE with associated immune MGN (lupus “full-house” deposits were not found on renal biopsy) and reactive lymphadenopathy had been proposed in 2008 and maintained until 2012 in view of the presence of anti-dsDNA, thrombocytopenia, arthritis and history of cutaneous rash. Other entities occasionally associated with high serum IgG4 level or with IgG4+ plasma cells on biopsy samples were also ruled out (i.e. granulomatosis with polyangiitis (GPA), Castleman’s disease or McDuffie hypocomplementemic urticarial vasculitis [4]).

IgG4-RD is considered as definite in this case because all three comprehensive diagnostic criteria are met: diffuse swelling in lymph nodes, elevated serum IgG4 concentration ≥ 135 mg/dL (2790 mg/dL in 2012) and lymph node infiltration of IgG4 + plasma cells with ratio of IgG4+/IgG+ cells > 40% and >10 IgG4+ plasma cells per HPF [5].

MGN are responsible for 30% of adult nephrotic syndromes. Eighty percent of MGN are idiopathic whereas the other 20% are caused by various pathologies which we searched for such as infections, cancers and systemic diseases. Circulating antibodies to the PLA2R (which is a transmembrane protein located on podocytes) are found in 70% of idiopathic membranous glomerulonephritis (IMGN). These antibodies are of IgG4 subclass but high serum IgG4 levels are not encountered in IMGN. The observation of glomerular deposits of PLA2R antibodies on biopsy seems to be more sensitive than their detection in serum. However, this could not be done here giving the absence of a recent renal biopsy [3, 6].

Kidney involvement in IgG4-RD or IgG4-RKD occurs in approximately 15% of IgG4-RD patients. It has the remarkable property to result in two well distinct types of lesions: first, TIN which is most commonly encountered and second, glomerulopathy. Pyelitis have also been described. Glomerular involvement is very rare (7% of IgG4-RKD). Lymphoplasmocytic infiltrate and storiform pattern of fibrosis need not be present for its diagnosis. MGN is the only glomerular lesion that can be reliably attributed to IgG4-RD. However, other forms of glomerulopathy such as mesangioproliferative glomerulonephritis, IgA nephropathy and membranoproliferative glomerulonephritis have also been described by Saeki et al. [7]. Usually, glomerular lesions coexist with TIN as in our patient’s first renal biopsy. Isolated glomerular involvement in IgG4-RKD is uncommon (none of the biopsies studied by Saeki et al. [7] and in four of the nine biopsies studied by Alexander et al. [8]). Unfortunately, in our case IgG subclass could not be determined retrospectively on the renal biopsies of 2004 and 2008 because of insufficient material. However, anti-PLA2R antibodies were negative in the blood which makes IMGN less likely and points towards IgG4-related glomerulonephritis. This is supported by the good response to GC, the associated TIN in the first renal biopsy, the documentation of IgG4-RD in lymph nodes and very high serum IgG4 levels [2, 3, 7–10]. Jindal et al. [11] have described a similar case report of IgG4-related MGN diagnosed through nephrotic syndrome and lymphadenopathy. In that case, an AIP was associated (which is more usual) and end-stage renal disease with need for dialysis could not be avoided despite GC and rituximab [11].

Specific diagnostic criteria exist for IgG4-RKD [12]. They were used for our patient and confirmed definite IgG4-RKD although further analyses could not be performed on kidney biopsy to assess the presence of IgG4+ plasma cells. Thus IgG4-related generalized lymphadenopathy and IgG4-related kidney disease with MGN and TIN are definite in this case report according to such criteria. Skin and joint involvement would have needed further investigations to be confirmed. Thrombocytopenia has never been reported before such a manifestation of IgG4-RD. Its etiology in this case report actually remains unclear.

### Discussion about treatment, follow-up and pathophysiology of IgG4-RD

All patients with active IgG4-RD and a subset of asymptomatic patients require treatment, sometimes urgently. GC are currently the main first-line treatment with initial good clinical and biological response, especially when a low degree of fibrosis is present. However, relapses are very common as seen in this case report and maintenance GC is often required after induction therapy. Patients who relapse for the first time after successful induction should be inducted again by GC. Introduction of steroid-sparing immunosuppressive agents should be considered for following relapses due to the frequency of side effects associated with long term GC therapy [13]. Mycophenolate mofetil, methotrexate or azathioprine have shown some efficacy in IgG4-related pancreatitis [1, 14]. Rituximab is one of the most promising treatment of relapsing IgG4-related diseases. Rituximab is an anti-CD20 chimeric antibody that acts by depleting CD20 positive B-lymphocytes. Good response has been seen even without initial or concomitant GC therapy and it allowed to discontinue GC in patients with steroid dependence in several published cases. Serum IgG4 level is also lowered by this treatment. Clinical and biological response to rituximab is maintained even after B cell reconstitution [1, 3, 15]. However, in the case reported by Jindal et al. [11], renal function did not improve after rituximab therapy perhaps because its late introduction. Further investigations are needed to assess the exact position and optimal regimen of rituximab, in IgG4-RKD [11].

It is interesting to note that the patient received CYP in 2008 for immune thrombocytopenia. This treatment was followed by 3 years without any manifestation of the IgG4-RD, in the absence of maintenance GC. CYP was also given with GC to a patient with IgG4-related TIN in Australia, and similarly allowed clinical and radiological remission as well as stabilization of renal failure [14]. Alexander et al. [8] have described a good response of proteinuria in one patient with IgG4-related MGN treated by prednisolone and CYP. More experience with CYP is required to confirm its effectiveness in IgG4-RD.

IgG4-RD follow-up is based on biological markers such as serum CH50, C3c, C4, IgG and IgG4 values. In case of relapsing IgG4-RD, a lowering of serum complement level is classically noticed (especially in IgG4-RKD) as well as increased IgG and IgG4 levels, as shown in Fig. 3 [2]. However, recent studies showed that these markers are poor predictors of the need for additional treatment. More attention should be paid to clinical manifestations [3]. PET/CT has a valuable role in mapping lesions, staging extent of the disease and guiding biopsy. Some studies showed that it could also be useful in monitoring response to treatment and identifying disease relapse earlier than other imaging techniques [4].

IgG4-RD pathophysiology is not completely understood yet. Excess of T helper 2 (Th2) cells and regulatory T (Treg) cells has been described in involved organs. This could explain fibrosis (since transforming growth factor-beta is secreted by Treg) and increased production of IgG4 (because cytokines secreted by Th2 and Treg stimulate IgG4 class switch). Excess of Th2 can also explain IgE excess and hyper eosinophilia [1, 16]. This immune imbalance could be due to abnormal reaction against pathogenic agents (i.e. Helicobacter pylori) or to commensals belonging to normal microbiota with continuous presentation of antigen by B cells. This could explain clinical improvement observed following B-cell depletion by rituximab [3]. In genetic studies, higher prevalence of HLA-DRB10405 and HLA-DQB10401 has been reported in Japanese patients with IgG4-RD [1].

An important question remains: are IgG4 pathogenic or not in IgG4-RD? The recent discovery of serum IgG4-negative IgG4-RD raises questions about the causative role of the IgG4 molecule [2]. ANA are frequently found in IgG4-RD patients as seen in this case report and are suspected to be pathogenic but do not belong to the IgG4 subclass [17]. Moreover, IgG4 is a relatively non-inflammatory antibody because it undergoes heavy-chain exchange leading to monovalent but bispecific antibody with reduced ability to crosslink antigens and form immune complexes. The amino acid sequence in its CH2 domain also limits IgG4 interaction with C1q and Fcγ receptors [3, 18]. Nevertheless, recent studies show the capacity of IgG4 to form aggregates which can cause inflammatory lesions in the absence of antigen [19]. So, IgG4-RD can be considered either as an allergic disease where IgG4 are only a marker of Th2 and Treg excess in tissues [1] or can be seen as an immune disorder where IgG4 could play by itself in the pathology [7].

## Conclusions

We have described the rare history of a patient with MGN secondary to IgG4-RD without pancreatitis.

Several mechanisms still remain to be discovered about IgG4-RD such as the role of IgG4 antibodies and even the nature of the pathology itself. However, it is now a better defined pathology with comprehensive diagnostic criteria and a large number of cases are now described.

GC therapy is a potent initial treatment in IgG4-RD, avoiding end-stage renal disease in IgG4-RKD for example. It must be initiated before occurrence of fibrosis for better response. This emphasizes the importance of early diagnosis. However, resistance and side effects to GC finally occur and steroid-sparing immunosuppressive agents have to be considered. Rituximab seems to be a promising treatment. CYP also proved to be a very good therapy in our case and in others: these case observations would justify the design of controlled clinical trials to evaluate its effectiveness in IgG4-RD.

IgG4-RD follow-up can be based on biological markers such as serum CH50, C3c, C4, IgG and IgG4 values, but they are imperfect predictors. PET/CT could be more reliable for the follow-up.

The differential diagnosis of IgG4-related generalized lymphadenopathy from lymphoproliferative disorders (especially malignant lymphoma) is very difficult. PET/CT is not helpful in this indication and lymph node biopsies are crucial to exclude hematologic malignancies. Diagnosis of IgG4-RD does not dispense to perform new lymph nodes biopsies when relapse occurs. Indeed, higher risk of developing lymphoma in IgG4-RD patients is suspected.

Kidney involvement is rare without associated pancreatitis. Glomerular involvement (mainly MGN) is even more uncommon especially without associated TIN. Further studies of MGN secondary to IgG4-RD will be welcome to improve our current knowledge of this disease. Nevertheless IgG4-related MGN appears already to be a well-established cause of MGN which must be sought after in every patient with a previous diagnosis of IgG4-RD but also in every patient with MGN found on renal biopsy.

## Abbreviations

AIP: Autoimmune pancreatitis
ANA: Anti-nuclear antibodies
CT: Computed tomography
CYP: Cyclophosphamide
GC: Glucocorticoids
HPF: High powered field
IgG4-RD: IgG4-related disease
IgG4-RKD: IgG4-related kidney disease
IL: Interleukin
IMGN: Idiopathic membranous glomerulonephritis
MGN: Membranous glomerulonephritis
MRI: Magnetic resonance imaging
PET/CT: Positron-emission tomography/computed tomography
PLA2R: Anti-phospholipase A2 receptor
SLE: Systemic lupus erythematous
Th2: T helper 2
TIN: Tubulo-interstitial nephropathy
Treg: Regulatory T
